# Delayed diagnosis of fatal pneumonic canine plague: clinical and pathologic features in two naturally infected Colorado dogs

**DOI:** 10.1186/s12917-020-02361-z

**Published:** 2020-05-25

**Authors:** Paula A. Schaffer, Connor S. Hershkowitz, Kristy L. Dowers, Jennifer L. Golchanour, Lauren J. Harris, Tawfik A. Aboellial, Paul S. Morley, Stephanie A. Brault, Kristy L. Pabilonia, Gary L. Mason, Jennifer A. House, Joshua B. Daniels

**Affiliations:** 1 Department of Microbiology, Immunology, and Pathology, College of Veterinary Medicine and Biomedical Sciences, Colorado State University, Fort Collins, CO USA; 2 Department of Clinical Sciences, College of Veterinary Medicine and Biomedical Sciences, Colorado State University, Fort Collins, CO USA; 3 Small Animal Specialist Hospital, Sydney, NSW Australia; 4 Living Springs Veterinary Care, LLC, Bennett, CO USA; 5 VERO - Veterinary Education, Research, and Outreach Program, Texas A&M University and West Texas A&M University, Canyon, TX USA; 6 National Animal Health Monitoring System (NAHMS), Animal and Plant Health Inspection Service (APHIS), USDA, Washington, D.C. USA; 7 Colorado Department of Public Health and Environment, Denver, CO USA

**Keywords:** Canine, Pneumonic plague, Yersinia pestis

## Abstract

**Background:**

Plague caused by *Yersinia pestis* is a highly infectious and potentially fatal zoonotic disease that can be spread by wild and domestic animals. In endemic areas of the northern hemisphere plague typically cycles from March to October, when flea vectors are active. Clinical forms of disease include bubonic, septicemic, and pneumonic plague. All clinical forms are uncommon in dogs and the pneumonic form is exceedingly rare.

**Case presentation:**

Two mixed breed young-adult male domestic dogs presented to Colorado veterinarians with fever and vague signs that progressed to hemoptysis within 24 h. Case 1 presented in June 2014, while Case 2 occurred in December 2017. Thoracic radiography of Case 1 and 2 revealed right dorsal and right accessory lobe consolidation, respectively. In Case 1 initial differential diagnoses included pulmonary contusion due to trauma or diphacinone toxicosis. Case 1 was euthanized ~ 24 h post presentation due to progressive dyspnea and hemoptysis. Plague was confirmed 9 days later, after the dog’s owner was hospitalized with pneumonia. Case 2 was treated as foreign body/aspiration pneumonia and underwent lung lobectomy at a veterinary teaching hospital. Case 2 was euthanized after 5 days of hospitalization when bacterial culture of the excised lobe yielded *Yersinia pestis*. Both dogs had severe diffuse necrohemorrhagic and suppurative pneumonia at post mortem examination.

**Conclusions:**

Both dogs were misdiagnosed due to the atypical lobar presentation of an extremely rare form of plague in a species that infrequently succumbs to clinical disease. Presentation outside of the typical transmission period of plague was also a factor leading to delayed diagnosis in Case 2. Erroneous identification by automated bacterial identification systems was problematic in both cases. In endemic areas, plague should be ruled out early in febrile dogs with acute respiratory signs, hemoptysis, lobar or diffuse pathology, and potential for exposure, regardless of season. Seasonal and geographic distributions of plague may shift with climate change, so vigilance by primary care veterinarians is warranted. Timely submission of samples to a veterinary diagnostic laboratory could expedite accurate diagnosis and reduce potential for human and domestic animal exposure.

## Background

*Yersinia pestis* is a gram-negative, facultative, nonspore-forming bacillus that is of major worldwide concern to public health where humans interface with wildlife. In the northern hemisphere, sylvatic plague cycles in spring, summer, and fall [1]. In North America plague is especially common in the four-corners region (Arizona, Colorado, New Mexico, and Utah) [1]. Rodents such as ground squirrels, rats, and mice serve as the typical reservoir hosts [1, 2]. Other species such as rabbits, skunk, mule deer, other wildlife, and domestic species can be infected when exposed to *Y. pestis* via flea vectors. Predators such as bobcats and cougars can also be infected when they ingest infected prey (oropharyngeal or respiratory exposure) [3–5]. Humans are exposed to the bacteria via bites from infected flea vectors, infection of cutaneous wounds (usually through contact with infected carcass without personal protective equipment), or via inhalation/ingestion of infective material.

Clinical plague may be classified into three classic forms: bubonic, septicemic, and pneumonic. The bubonic form follows percutaneous exposure and produces multicentric necrosuppurative lymphadenitis with numerous bacterial colonies, vasculitis, thrombosis, and hemorrhage. The septicemic form is characterized by bacteremia with multiorgan inflammation and necrosis. The pneumonic form is characterized by diffuse necrotizing pneumonia of the lower respiratory tract and can be contagious through aerosolization [6].

Domestic cats present a heightened public health risk because they may hunt infected species (rodents, rabbits) and are prone to the pneumonic form of the disease, which is highly contagious [7]. Cat-associated human plague cases were responsible for 7.7% of the total human cases in the Western U.S. and for five deaths between 1977 and 1998 [7].

By contrast, clinical plague is very rare in dogs, even in endemic areas where dogs may be regularly exposed to the bacteria [8]. Dogs develop antibody titers in plague-endemic regions and during outbreaks [9–13]. In one study, antibody titers were detected in 41% of dogs sero-surveyed during a rodent plague outbreak in California [14]. When dogs do become clinically ill with plague, they typically present with the bubonic or septicemic forms [8, 10], which have a relatively low risk of direct transmission. Domestic dogs have been occasionally implicated in the transmission of plague to humans through carriage of infected fleas resulting in the bubonic form of disease [15, 16].

We describe the clinical course and pathology of two unusual cases of canine pneumonic plague. Both potentially exposed over 100 people; one resulted in illness in four humans. Veterinarians have a key role in plague identification and can serve as the first defense for people in endemic areas. They are also among the humans most at risk for zoonotic transmission of plague [8, 17, 18]. Rapid diagnosis of canine pneumonic plague is critically important in order to prevent morbidity and mortality in potentially exposed humans and other domestic animal species.

## Case 1 presentation

In June of 2014, a 2 year old neutered male domestic mixed breed dog was presented to a Colorado veterinarian for evaluation of rigid jaw tone, drooling, pale mucous membranes, and right front limb lameness. The dog was up to date on routine vaccines and had no significant prior medical history. Upon examination, the dog was febrile (106 ° F) with a mild leukopenia (5320 cells μL, reference range [RR] 6000–17,000 cells/μL) characterized by a monocytopenia (30 cells/μL, RR 200–1500 cells/μL) and borderline neutropenia (2960 cells/μL, RR 3000-12,000 cells/μL). Erythrogram data were unremarkable. The patient was thrombocytopenic (56,000 cells/μL, RR 200,000-500,000 cells/μL). The biochemistry panel revealed mild hypoalbuminemia (2.2 g/dL, RR 2.5–44 g/dL), mildly elevated ALP (207 IU/L, RR 20–150 IU/L), moderate hyponatremia (131 mmol/L, RR 138–160 mmol/L) and mild hypokalemia (3.6 mmol/L, RR 3.7–5.8 mmol/L).

Thoracic radiographs showed opacity of the right dorsal lung lobe. The primary differential diagnoses included pulmonary contusion due to a kick from a horse or diphacinone toxicity. A prairie dog colony on the owner’s property had been depopulated with diphacinone 8–10 months prior to the dog’s illness, and the owner recalled seeing deceased prairie dogs dug up on the property in the months preceding the dog’s illness. The dog was admitted and treated with IV fluids (Lactated Ringers solution, 80 mL/kg/hr for 3 h then maintenance rate 60 mg/kg/day), Vitamin K-1 (5 mg/kg IM Q12 h), flunixin meglumine (1 mg/kg SQ), activated charcoal by mouth (50 mL once), maropitant citrate (1 mg/kg SQ, Q12 h) and dexamethasone sodium phosphate (1 mg/kg IM, Q24 h). He was humanely euthanized approximately 24 h after presentation due to progressive dyspnea and hemoptysis.

The dog’s carcass was submitted to the Colorado State University Veterinary Diagnostic Laboratory (CSU VDL) for post-mortem examination. Gross pathologic changes were dominated by extensive hemorrhage in all lung lobes, frank blood in the gastrointestinal tract, and ecchymotic to volume hemorrhages of the mediastinum, pleura, and diaphragm. No anticoagulants were detected in liver tested by high performance liquid chromatography. The dog tested negative for rabies by immunofluorescence assay. Histopathology was initially declined by the owner.

One of the dog’s two owners was hospitalized with acute pneumonia 4 days after the dog’s euthanasia. The owner’s medical team contacted the CSU VDL approximately 5 days after the owner’s admission to the hospital and reported that the owner had an acute undiagnosed respiratory disease and to ascertain the cause of the dog’s respiratory illness. *Yersinia pestis* was subsequently confirmed in the owner of the dog by culture and real-time PCR. Frozen archived tissues from the dog (lung, liver, kidney, small intestine, stomach content, brain, and whole blood) were tested by real-time PCR for *Yersinia pestis* and *Francisella tularensis* at the CSU VDL and *Y. pestis* was detected in all samples. Samples were then submitted to the Centers for Disease Control and Prevention and were confirmed positive for *Y. pestis.*

Formalin fixed tissues from the dog’s necropsy were processed for review. Histologically, there was severe acute necrosuppurative bronchopneumonia with very large numbers of intra-alveolar bacteria (Fig. 2a) and widespread hemorrhage. Low numbers of small and randomly scattered fibrinosuppurative foci were identified in the spleen and meninges, indicating disseminated disease at the time of death.

Contact with case 1 resulted in four confirmed human cases of plague. The dog’s owner and a close contact were both hospitalized due to pneumonia. Two workers at the veterinary clinic acquired febrile illness with cough and chest tightness. All four seroconverted to *Y. pestis* and recovered after medical intervention. A total of 114 additional persons had potential exposure from the dog (36 people) or one of the human patients (78 people). The human medical perspective and public health investigation and response are reported elsewhere [18, 19].

## Case 2 presentation

A 3 yr old mixed breed dog was presented in December 2017 to a Colorado veterinarian for an 8-h history of lethargy, anorexia, and trembling that had been preceded by 2 days of mild cough. The dog was up to date on vaccines. The owner reported jogging with the dog near prairie dog colonies where a dead prairie dog had been spotted. Physical examination was normal except for hyperthermia (103.1 °F). Complete blood count (CBC) revealed leukocytosis (23,400 cells/μL, RR 5050–16,760 cells/μL) with neutrophilia (18,920 cells/μL, RR 2.6–11.0 × 10^3^ cells/μL). Serum biochemistry analysis found mild elevations in ALT (145 IU/L, RR 10–125 U/L) and GGT (17 IU/L, RR 0–11 IU/L). Concerns for canine infectious respiratory disease complex agents, bacterial hepatitis, leptospirosis, plague, or another infectious etiology were raised. Hospitalization for supportive care, WITNESS Lepto antibody test^a^, and plague screening were declined by the owner in favor of subcutaneous fluids (25 mL/kg once), maropitant citrate (1 mg/kg SQ once), and amoxicillin/clavulonic acid (17.8 mg/kg PO Q12 h for 7 days) and instructions to recheck with the primary veterinarian in 2–4 days. The next day, the dog returned with progressive lethargy, anorexia, and hemoptysis. Physical examination revealed worsening hyperthermia (104.5 °F), dehydration, and increased bronchovesicular sounds; no cough was noted or elicited. Thoracic radiographs, PT/aPTT, and leptospirosis testing were declined in favor of referral to the Colorado State University Veterinary Teaching Hospital (VTH).

On presentation to the VTH (Day 0), the dog was febrile (105.1 °F), tachypneic (72 brpm) with normal lung sounds, and had a prolonged capillary refill time (3 s). Venous blood gas revealed a metabolic acidosis: pH 7.294 (RR 7.33–7.45), cHCO3 14.0 mEq/L (RR 17–27 mEq/L), hyperlactatemia 3.2 mmol/L (RR 0.20–1.44 mmol/L). No cough was appreciated. Thoracic Focused Assessment with Sonogram for Trauma (T-FAST) for detection of pleural and pericardial effusion was negative. Repeat CBC found moderate neutropenia (1700 cells/μL, RR 2600-11,000 cells/μL) with bands (200 cells/μL, RR 0–200 cells/μL) and moderate toxic change, lymphopenia (300 cells/μL, RR 1000–4800 cells/μL), and severe thrombocytopenia (48,000 cells/μL, RR 200,000-500,000 cells/μL) with few clumps and a normal MPV (8.6 fL, RR 7.5–14.6 fL). Urinalysis was unremarkable. SNAP 4Dx^d^ for *Ehrlichia* spp., *Anaplasma* spp., *Borrelia burdorferi* C_6_ antibodies, and heartworm antigen was negative.

**Fig. 1:**
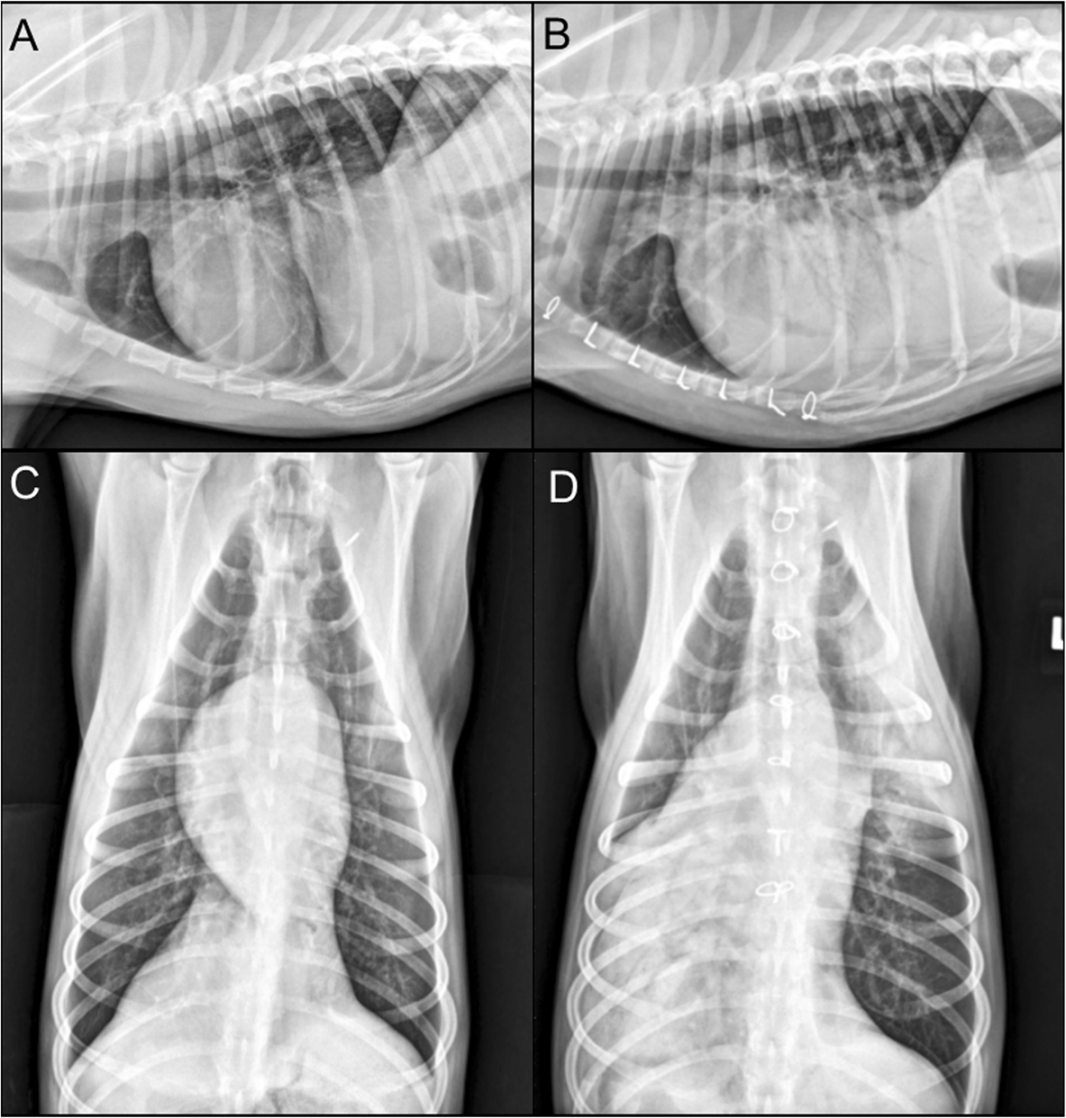
Thoracic radiography of a dog with pneumonic plague (case 2). Left lateral (**a**) and ventral-dorsal (**c**) imaging of the thorax upon presentation to the veterinary teaching hospital demonstrate poorly defined alveolar patterns in the right accessory lung lobe. In radiographs 5 days later (post accessory lobectomy) multiple lobes have patchy to lobar alveolar patterns (**b**) and (**d**)

Thoracic radiographs showed an alveolar pattern limited to the accessory lung lobe (Fig. 1a and c). This was interpreted as bronchopneumonia, possibly consistent with aspirated or migrating foreign body. This interpretation was supported by the dog’s young age, dolichocephalic confirmation, and frequent outdoor activities that had resulted in a previous episode of grass awn aspiration, according to the owner. The dog was hospitalized overnight with IV fluids, ampicillin sodium/sulbactam sodium (50 mg/kg IV Q8 h), enrofloxacin (10 mg/kg IV Q24 h), maropitant citrate (1 mg/kg IV Q24 h), and nebulization (Q6 h). Coughing became apparent overnight.

On Day 1 of hospitalization, the patient had diffuse harsh lung sounds and hemoptysis. There was mild hypoglycemia (64 mg/dL, RR 70–115 mg/dL) and hypoproteinemia (4.7 g/dL, RR 5.0–7.0 g/dL; hypoalbuminemia 2.5 g/dL, RR 3.0–4.3 g/dL; normoglobulinemia 2.2 g/dL, RR 1.5–3.2 g/dL) as well as moderate hyperbilirubinemia (0.5 mg/dL, RR 0.0–0.2 mg/dL), suspicious for sepsis. Mildly elevated ALP (283 IU/L, RR 15–140 IU/L), moderately elevated creatine kinase (1599 IU/L, RR 50–275 IU/L), mild hypobicarbonatemia (11.4 mEq/L, RR 15–25 mEq/L), and hypocalcemia (8.6 mg/dL, RR 9.0–11.5 mg/dL) were also noted. Coagulation panel was concerning for early disseminated intravascular coagulation due to mild prolongation of aPTT (18.0 s, RR 9.8–13.3 s), high end of normal PT (9.2 s, RR 7.4–9.4 s), elevated fibrin degradation products (5.0 μg/mL, RR 0–4.0 μg/mL), elevated D-dimers (0.48 μg/mL, RR 0.03–0.40 μg/mL), low antithrombin III (79%, RR 104–162%), and elevated quantitative fibrinogen (594 mg/dL; 123–210 mg/dL). Computed tomography scan in preparation for a median sternotomy and lobectomy confirmed a consolidated accessory lung lobe interpreted as consistent with foreign body abscessation, multifocal interstitial and alveolar patterns in the remaining lobes, and sternal, cranial mediastinal, and tracheobronchial lymphadenopathy interpreted as reactive.

Median sternotomy, accessory lung lobectomy, and chest tube placement were performed without complication. At surgery, the accessory lung lobe was diffusely consolidated with multifocal to coalescing well-defined regions of hemorrhage on all surfaces. There were scattered small red foci in all other lobes, likely corresponding to the multifocal interstitial and alveolar patterns noted on imaging, which were interpreted as small sites of atelectasis. Scant serosanguinous effusion was noted. Samples of free fluid and a swab of incised lung surface were submitted for bacterial culture and sensitivity. The accessory lung lobe was submitted for histopathology.

The patient recovered in an oxygen cage (30–40%) with fentanyl (2–5 mcg/kg/hr), ketamine (3 mcg/kg/min), intrathoracic lidocaine (1 mg/kg Q8 h), and intrathoracic bupivacaine (1 mg/kg Q8 h) provided. The chest tube was evacuated routinely. All previous medications were continued. Overnight the patient vomited once, but was normothermic and eupneic with an oxygen index greater than 300.

On the following day (Day 2 of hospitalization), oxygen therapy was discontinued and the chest tube was removed. Oral analgesics (tramadol 50 mg PO Q8 h; gabapentin 100 mg PO Q8 h) replaced ketamine and fentanyl. Overnight, the patient became transiently febrile (up to 104 °F). Mild tachypnea (60 brpm), inspiratory dyspnea, diffuse harsh lung sounds, and right-sided crackles developed. Oxygen therapy (40%) was reinstituted. Ondansetron (0.5 mg/kg IV Q12 h) and pantoprazole (1 mg/kg IV Q12 h) were initiated for inappetence and nausea. Repeat CBC found a resolved neutropenia (9,800 cells/μL, RR 2600-11,000 cells/μL) with moderate toxic changes and no left shift, few reactive lymphocytes, and worsening thrombocytopenia (27,000 cells/μL, RR 200,000-500,000 cells/μL) with absent clumps and evidence of increased production (MPV 20.4 fI, RR 7.5–14.6 fI; moderate giant platelets). Repeat thoracic radiographs (Fig. 1b and d) found a progressive multifocal interstitial pattern in all lobes and pleural fissure lines.

**Fig. 2:**
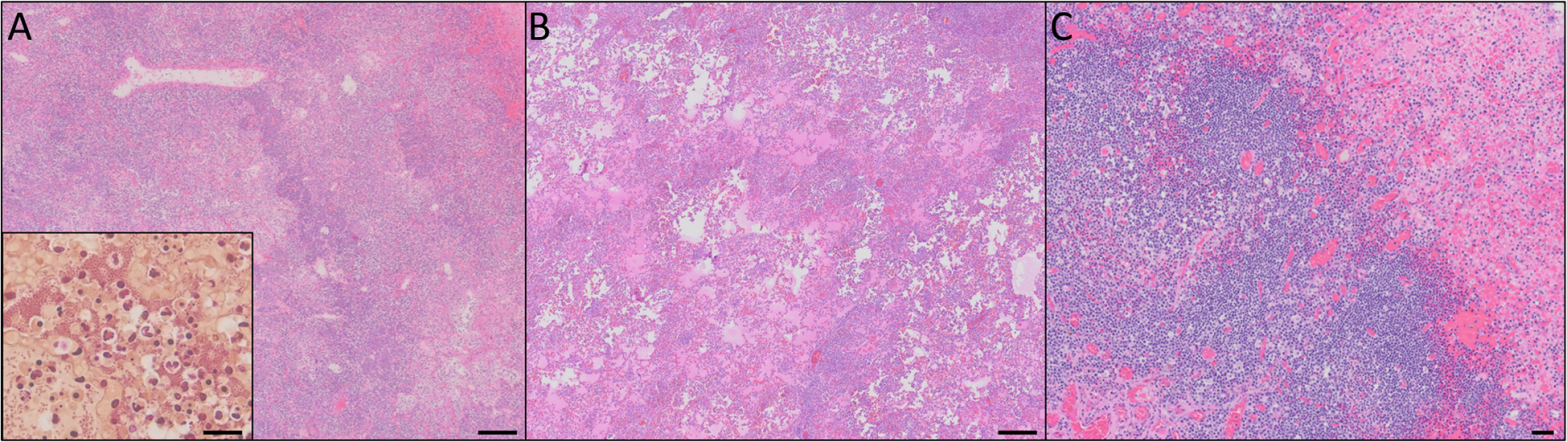
Histopathology of pneumonic plague in two dogs. **a** Case 1 with diffuse severe necrohemorrhagic pneumonia and large numbers of intralesional bacteria (inset, gram stain) and **b** Case 2 with similar pathology but no intralesional bacteria. **c** Case 2 had severe acute necrotizing tonsillitis. Haematoxylin and eosin stain; bar = 200 μm for panels A and B; 50 μm for panel C, and 20 μm for inset

Histopathology was read by a board-certified veterinary pathologist on the afternoon of Day 3 of hospitalization (Fig. 2). The parenchyma was effaced by extensive areas of necrosis and hemorrhage with infiltration by large numbers of neutrophils and fewer foamy alveolar histiocytes. In less severely affected areas, alveolar septae were thickened by edema and fibrin. The pleural surface was lined by a mat of fibrin and degenerate neutrophils. Bacteria were not identified with routine hematoxylin and eosin or in a Gram-stained preparation. A diagnosis of severe suppurative and necrohemorrhagic bronchopneumonia with fibrinopurulent pleuritis was made, consistent with a highly pathogenic bacterial infection. *Y. pestis* was not specifically considered by the pathologist, due to the lack of intralesional coccobacilli, a typical feature of plague infection [20]. Doxycycline (5 mg/kg IV Q12 h) was added and ampicillin-sulbactam/enrofloxacin continued.

On Day 4 of hospitalization, the dog was normothermic but harsh lung sounds persisted. Tramadol was discontinued and capromorelin (40.2 mg PO Q24 h) and cisapride (5 mg PO Q8 h) were initiated due to inappetence. Due to concerns that additional abscesses may be causing bacteremia, an abdominal ultrasound was performed and was unremarkable. On the night of Day 4, the patient remained stable, showed interest in food, and remained normothermic.

On Day 5, the patient became febrile (103.6 °F to 104.5 °F) and dyspneic with bilaterally severe harsh lung sounds and significant right-sided crackles. Repeat CBC showed worsening neutrophilia (11,600 cells/μL, RR 2600-11,000 cells/μL) with toxic changes, bands (100 cells/μL, RR 0–200 cells/μL), and acanthocytes, consistent with fragmentation injury from DIC. Serum biochemistry panel found worsening hypoproteinemia (3.6 g/dL, RR 5.0–7.0 g/dL; hypoalbuminemia 1.7 g/dL, RR 3.0–4.3 g/dL; normoglobulinemia 1.9 g/dL, RR 1.5–3.2 g/dL), hypocalcemia (7.5 mg/dL, RR 9.0–11.5 mg/dL), and hypomagnesemia (1.4 mg/dL, RR 1.8–2.4 mg/dL) with hypokalemia (3.45 mEq/L, RR 3.9–5.4 mEq/L).

Aerobic culture of lung, sampled intraoperatively, yielded sparse, pure growth of a gram-negative organism, which was initially identified as *Yersinia pseudotuberculosis* by matrix-assisted laser desorption/ionization time-of-flight mass spectrometry (MALDI-TOF). This was considered suspicious when reviewed by a veterinary clinical microbiologist due to the body site (lung) from which the organism was isolated, as well as the inconsistency of *Y. pseudotuberculosis* with the clinical picture. The bacterial isolate and original lung and pleural fluid swab samples were tested for *Y. pestis* by real-time PCR and *Y. pestis* was detected in these samples. Biosecurity measures were implemented throughout the VTH. The owner subsequently elected humane euthanasia.

A limited necropsy was performed by a board-certified veterinary pathologist in a specialized BSL2 necropsy facility. Both lungs were multifocally consolidated, especially the cranial lung lobes. Severely affected areas released large amounts of serosanguineous fluid when incised. Diaphragmatic lung lobes failed to collapse and were moderately to markedly hemorrhagic. Tonsils, submandibular, tracheobronchial and mediastinal lymph nodes were mildly to moderately enlarged and edematous. Samples of the tonsils, lungs, spleen, lymph nodes, small intestine, and kidneys were collected and processed routinely for histologic examination. In addition to diffuse severe necrosuppurative and hemorrhagic pneumonia there was acute moderate necrosuppurative tonsillitis (Fig. 2c). Tonsillar lymphoid follicles showed prominent lymphocytolysis and expansion of interlobular septa by neutrophils. No histologic lesions were present within the spleen, small intestine, or lymph nodes. *Y. pestis* was detected in a post mortem sample of liver by real-time PCR; tonsil, lung, spleen, lymph node, small intestine, and kidney samples were negative.

Case 2 resulted in potential exposure at the VTH of 116 people including veterinarians, veterinary support staff and laboratory technicians, veterinary students, and volunteers and potential exposure of 46 other animals. No individuals were confirmed to have contracted plague. The public health response and ramifications are reported elsewhere [21].

## Discussion and conclusion

Pneumonic plague is very uncommon in dogs with only one previous case reported as the source of a human outbreak in China [22]. Most dogs with clinical plague present with the septicemic or bubonic plague and may have fever, lymphadenopathy, anorexia, vomiting, diarrhea, and peripheral abscesses [8, 10]. In one study of 62 canine plague cases from New Mexico [8], only 9 dogs (15%) had vague respiratory signs recorded at presentation such as harsh lung sounds and increased respiratory effort. Thoracic radiography was only pursued in 1 patient, which revealed a “bronchial pattern” not necessarily consistent with pneumonic plague. Most dogs were treated with antibiotics and sixty (97%) survived without experiencing notable pulmonary disease. Only one dog died with respiratory signs described as tachypnea and dyspnea, but post mortem findings were not reported and the cause was thus not confirmed.

In contrast, the two dogs described here presented with fever, malaise, and lobar pneumonia that quickly progressed to hemoptysis within 24 h and they had diffuse pneumonia at necropsy, consistent with pneumonic plague. Hemoptysis is a relatively rare finding in dogs; a 9 year retrospective study from a large referral hospital found only 36 dogs with hemoptysis as a presenting complaint [23]. Notably, hemoptysis was present in the two dogs described here as well as the case of canine pneumonic plague in China [22]. This clinical sign, though not specific, should prompt testing for plague in a febrile pneumonic dog in endemic areas.

The preferred diagnostic specimens for plague are, in order, 1) aspirate of enlarged lymph node or buboe for PCR, 2) whole blood for PCR, 3) fresh tissue for PCR, 4) blood culture, and 5) serology (single high titer or paired titers) [3]. Peripheral lymphadenopathy was not present in either case described here. In retrospect, whole blood for PCR or culture could have been pursued, but it is unknown if these would have been diagnostic. Not all septic cases are bacteremic, and there is no published data, to our knowledge, regarding the sensitivity of blood culture in canine pneumonic plague. Antibiotic therapy in case 2 may have also reduced the likelihood of a positive blood sample by either PCR or culture.

Gross findings in both dogs included severe diffuse pulmonary hemorrhage. Given the systemic hemorrhage noted at post mortem, this was initially interpreted in Case 1 as consistent with rodenticide toxicity. Case 2 initially presented with lobar consolidation that was interpreted as consolidated lung lobe secondary to migrating foreign body with abscessation. At post mortem examination both cases had progressed to diffuse hemorrhagic pneumonia. These two cases suggest that pneumonic plague should be a differential diagnosis for gross lesions ranging from lobar to diffuse pulmonary hemorrhage and/or hemorrhagic necrotizing to necrosuppurative pneumonia. Consequently, pneumonic plague should be considered whenever anticoagulant rodenticide, pulmonary contusion, aspiration pneumonia/bronchopneumonia, or lung lobe abscessation secondary to foreign body inhalation/migration are suspected, especially in endemic areas when there are pertinent historical factors such as wildlife or flea exposure.

Histologically both cases had severe, acute, diffuse, necrohemorrhagic and suppurative pneumonia. Case 1, which had not been treated with antibiotics, had florid bacteria evident in histologic sections of lung, which is typical of pneumonic plague in most species [20], and the bacteria were detected in multiple samples by real-time PCR (lung, liver, kidney, small intestine, stomach content, brain, and whole blood). Case 2, which had received 3 doses of an ampicillin/sulbactam and 1 dose of enrofloxacin prior to lung lobectomy, did not have histologically detectable bacteria in the excised lobe. The absence of visible bacteria in histologic lung sections may have been due to antimicrobial administration. This hypothesis is supported by the fact that the surgical lung swab yielded only light growth (fewer than 10 discrete colonies). No bacteria were identified histologically in any examined post mortem tissue (following 5 days of enrofloxacin therapy and ~ 2 days of doxycycline), and real-time PCR positivity of tissue at post mortem was limited to liver. Findings of this case highlight the importance of considering plague in canine patients with necrohemorrhagic to necrosuppurative pneumonia when antibiotics have been administered, and also emphasize the importance of collecting diagnostic samples prior to administration of antimicrobial therapy.

The bacterial isolate from Case 2 and an isolate from the initial human case associated with Case 1 were both initially misidentified by routine laboratory testing methods, as described in detail elsewhere [18, 21]. In previous reports *Y. pestis* has been misidentified as *Y. pseudotuberculosis, Pseudomonas luteola,* and *Acinetobacter lwoffii* [24]. Importantly, these misidentifications may not be recognized if a microbiologist does not perform secondary evaluation and interpretation of automated results. When plague is a concern, clinicians should submit specimens to a qualified veterinary diagnostic laboratory or public health laboratory to ensure accurate diagnosis. Further testing may be necessary when faced with questionable automated results from animals with signs compatible with plague. Commercial laboratories may have to forward samples to qualified reference laboratories, a process that delays accurate diagnosis.

Case 2 presented in December of 2017, outside the typical period of plague transmission (March to October) in the Northern Hemisphere. Higher temperatures and decreased snowfall in 2017 may have kept rodents and flea vectors active. Importantly, climatic shifts may alter both the seasonal range and the geographic distribution of plague infection [25, 26]. Continued encroachment of urban development into wildlife areas may also increase the potential for interface between the sylvatic cycles and domestic animal species and human beings. Practicing a “One Health” approach will heighten awareness of this potential among veterinarians, public health professionals, and the public and will be key to preventing exposures.

In summary, canine pneumonic plague is a rare but extremely important differential diagnosis for dogs with fever and acute respiratory signs, especially hemoptysis, in endemic areas. Importantly, this disease can present initially as lobar pneumonia, leading to differential diagnoses of trauma, anticoagulant-associated hemorrhage, aspiration/bronchopneumonia, or lung lobe abscessation secondary to foreign body inhalation. Automated bacterial identification may not accurately recognize *Yersinia pestis*, so cautions interpretation is warranted when plague is suspected. Delay in an accurate diagnosis for Case 1 resulted in 118 potential human exposures and four confirmed cases of human plague with two hospitalizations. Delay for Case 2 resulted in at least 116 potential human and 46 potential animal exposures with no reported morbidity. In China, a similar case of canine pneumonic plague was responsible for infection of 12 people, three of whom died [22]. The two cases presented here highlight several challenges that can impact timely diagnosis of this serious disease. Increased awareness of the clinical features of pneumonic plague in dogs may help to prevent misdiagnosis and unintended human and veterinary exposures in the future.

## Data Availability

The authors declare that all data supporting the findings of this study are available within the article. No datasets were generated or analyzed during the current study.

## Abbreviations

CBC: Complete blood count
CSU VDL: Colorado State University Veterinary Diagnostic Laboratory
RR: Reference range
T-FAST: Thoracic focal assessment with sonography for trauma
VTH: Veterinary Teaching Hospital
